# Ablação por Radiofrequência para Tratamento de Arritmias Cardíacas no Brasil: O Cenário do Sistema Único de Saúde

**DOI:** 10.36660/abc.20250411

**Published:** 2026-05-21

**Authors:** Ricardo Alkmim Teixeira, Elerson Arfelli, Fatima Dumas Cintra, Jose Carlos Moura Jorge, Alexsandro Alves Fagundes, Martino Martinelli

**Affiliations:** 1 Universidade do Vale do Sapucaí Pouso Alegre MG Brasil Universidade do Vale do Sapucaí – UNIVAS, Pouso Alegre, MG – Brasil; 2 Universidade de São Paulo Hospital das Clínicas da Faculdade de Medicina de Ribeirão Preto Ribeirão Preto SP Brasil Universidade de São Paulo Hospital das Clínicas da Faculdade de Medicina de Ribeirão Preto, Ribeirão Preto, SP – Brasil; 3 Universidade Federal de São Paulo São Paulo SP Brasil Universidade Federal de São Paulo, São Paulo, SP – Brasil; 4 Irmandade da Santa Casa de Misericórdia de Curitiba Curitiba PR Brasil Irmandade da Santa Casa de Misericórdia de Curitiba, Curitiba, PR – Brasil; 5 Pontifícia Universidade Católica do Paraná Curitiba PR Brasil Pontifícia Universidade Católica do Paraná, Curitiba, PR – Brasil; 6 Universidade do Estado da Bahia Salvador BA Brasil Universidade do Estado da Bahia, Salvador, BA – Brasil; 7 Instituto do Coração do Hospital das Clínicas da Faculdade de Medicina da Universidade de São Paulo São Paulo SP Brasil Instituto do Coração do Hospital das Clínicas da Faculdade de Medicina da Universidade de São Paulo, São Paulo, SP – Brasil

**Keywords:** Arritmias Cardíacas, Ablação por Radiofrequência, Barreiras ao Acesso aos Cuidados de Saúde, Sistema Único de Saúde

## Abstract

**Fundamento:**

As arritmias cardíacas comprometem a qualidade de vida e estão associadas a maior morbimortalidade. O tratamento farmacológico, em geral, tem eficácia limitada e efeitos adversos frequentes. A ablação por radiofrequência é eficaz e indicada para tratar diversos tipos de arritmias; entretanto, esta modalidade terapêutica não está amplamente disponível no Brasil pelo Sistema Único de Saúde (SUS).

**Objetivo:**

Analisar os procedimentos de ablação para o tratamento de arritmias cardíacas realizados no Brasil pelo SUS em 2019, a fim de identificar aspectos que limitam a disponibilidade desse tratamento aos pacientes do SUS.

**Métodos:**

Estudo retrospectivo com análise de dados do Sistema de Informações Hospitalares (SIH/SUS) e do Cadastro Nacional de Estabelecimentos de Saúde (CNES). Os procedimentos foram classificados por complexidade. A infraestrutura dos centros habilitados foi avaliada com base na disponibilidade dos equipamentos necessários e de profissionais capacitados.

**Resultados:**

Em 2019, foram realizados 6.471 procedimentos de eletrofisiologia pelo SUS no Brasil, sendo 82% de baixa ou média complexidade (grupos 1 a 3). As regiões Sul e Sudeste concentraram a maior parte dos procedimentos (48% e 31%, respectivamente). Apenas 53% dos centros possuíam o registro de todos os equipamentos necessários. O custo dos materiais excedeu de 1,5 a 4,8 vezes o valor reembolsado pelo SUS.

**Conclusões:**

No âmbito do SUS, existe disparidade regional significativa na disponibilização de serviços de eletrofisiologia, especialmente nas regiões Norte e Nordeste. A insuficiência de centros equipados e o baixo reembolso dos procedimentos são desafios críticos para melhorar o acesso ao tratamento de arritmias no Brasil.

**Figure:**
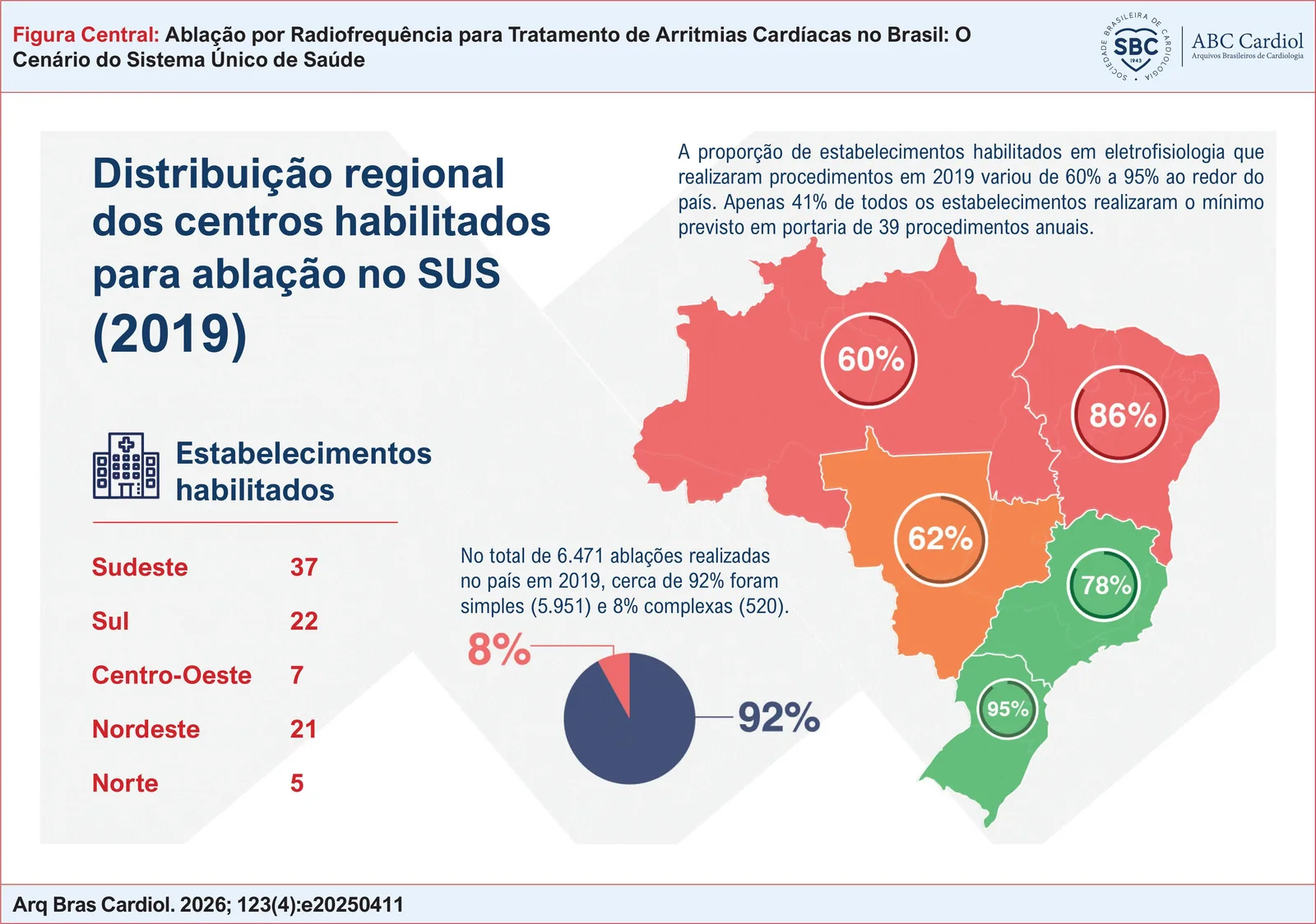


## Introdução

O Sistema Único de Saúde (SUS), um dos maiores sistemas públicos de saúde do mundo, oferece cobertura universal para toda a população brasileira. Em 2019, o SUS era o sistema de saúde exclusivo para 76% da população brasileira, estimada em 210 milhões de pessoas.^1^

De acordo com a Estatística Cardiovascular – Brasil 2020, elaborada pela Sociedade Brasileira de Cardiologia, as doenças cardiovasculares geram os maiores custos com hospitalização pelo SUS e representam a principal causa de concessão de pensões por incapacidade, além de serem a principal causa de morbidade. Neste cenário, a fibrilação atrial, arritmia cardíaca sustentada mais frequente, causou 321.866 hospitalizações (14,7 por 100 mil habitantes) pelo SUS entre 2008 e 2018^1^ e foi responsável por um custo de cerca de R$ 3,9 bilhões em 2015.^2^

O progresso tecnológico, combinado com uma compreensão mais detalhada dos mecanismos fisiopatológicos das arritmias, permitiu o desenvolvimento de métodos de diagnóstico mais precisos e tratamentos mais eficazes.^3^ Neste contexto, a ablação por cateter busca eliminar áreas pequenas de tecido cardíaco anormal que possibilitam a ocorrência de distúrbios elétricos no coração. Esta técnica é eficaz no tratamento de várias formas de arritmias, reduzindo a necessidade de medicação contínua e melhorando a qualidade de vida dos pacientes.^3^

Sendo um país em desenvolvimento, de dimensões continentais e de grande heterogeneidade socioeconômica, o Brasil apresenta muitas lacunas e diferenças regionais na assistência médica, inclusive no que se refere ao acesso às melhores estratégias para tratamento das arritmias cardíacas, em especial para as pessoas que dependem do SUS. Destaca-se ainda que atualmente o SUS não prevê remuneração para o tratamento de arritmias complexas (fibrilação atrial, taquicardia atrial cicatricial e arritmias ventriculares) por meio da técnica de referência atual (mapeamento eletroanatômico e cateter com força de contato). Neste sentido, recentemente a Comissão Nacional de Incorporação de Tecnologias no SUS (CONITEC), motivada por uma solicitação da Sociedade Brasileira de Arritmias Cardíacas (SOBRAC), emitiu um parecer favorável à incorporação do mapeamento eletroanatômico e cateter com força de contato para ablação de arritmias complexas pelo SUS.^4^ Entretanto, há escassez de dados estruturados acerca da utilização de técnicas modernas e resultados clínicos na perspectiva da saúde pública brasileira.^5^

Dessa forma, o objetivo deste estudo foi analisar o cenário nacional do tratamento de arritmias cardíacas no âmbito do SUS, com base no ano de 2019, devido à pandemia de COVID-19, que se manteve até o início de 2023.

## Métodos

Este estudo retrospectivo de abordagem quantitativa foi realizado a partir das bases de dados do Departamento de Tecnologia da Informação do Sistema Único de Saúde (DATASUS), compostas por informações de todo o país reportadas por documentos oficiais, como autorização de internação, dispensação de medicamentos e registros de óbito e nascimento.

Analisaram-se dois sistemas: a base de dados reduzida do Sistema de Informações Hospitalares do DATASUS (SIH/SUS), alimentada pela Autorização de Informação Hospitalar (AIH), e a base do Cadastro Nacional de Estabelecimentos de Saúde (CNES), sistema de informação oficial de cadastramento de informações de todos os estabelecimentos de saúde no país.^6^ Os dados extraídos da base de dados foram utilizados em sua forma original; não houve inclusão de dados faltantes ou exclusão de dados.

A geração da AIH ocorre no momento da internação em um prestador público ou privado conveniado ao SUS e enviada ao gestor da unidade prestadora de serviços. Mensalmente, os gestores enviam um arquivo magnético com os dados de todas as internações ocorridas no Brasil ao Ministério da Saúde para que se viabilize o faturamento dos serviços hospitalares prestados no SUS.^6^

Os dados da base do SIH/SUS foram extraídos com filtros relacionados ao diagnóstico, de acordo com a 10^a^ Revisão da Classificação Internacional de Doenças (Tabela S1), e ao procedimento realizado (Tabela S2).

A análise da base do CNES ocorreu por meio da seleção das habilitações 801 – Unidade de Assistência de Alta Complexidade Cardiovascular, 802 – Centro de Referência em Alta Complexidade Cardiovascular e 807 – Laboratório de Eletrofisiologia, sendo a última necessária com pelo menos uma das duas primeiras (801 ou 802). Os médicos cardiologistas que atuavam nos estabelecimentos analisados pela base do CNES deveriam estar listados na relação de habilitados na área de atuação em eletrofisiologia disponibilizada no site da SOBRAC (www.sobrac.org).^7,8^

Os equipamentos necessários para realização dos procedimentos foram definidos junto ao comitê da SOBRAC e em consonância com o disposto na Portaria N° 210 de 15 de junho de 2004 da Secretaria de Atenção à Saúde.^9^ Os equipamentos necessários estão dispostos na Tabela S3. O polígrafo, o mapeamento tridimensional e o gerador de radiofrequência não foram incluídos, já que podem ser portáteis e levados ao estabelecimento no dia do procedimento pelos fornecedores dos materiais.

Os procedimentos foram divididos em grupos conforme sua complexidade para análise (Tabela 1).

**Tabela 1:** Grupos de procedimentos utilizados nas análises

Estudo eletrofisiológico		Materiais compatíveis*
406050015	Estudo eletrofisiológico diagnóstico	2 introdutores (R$ 97)
**Ablação simples de circuitos em átrio direito**		
406050040	Estudo eletrofisiológico terapêutico I	2 introdutores (R$ 97)
406050023	Estudo eletrofisiológico terapêutico I (ablação de flutter atrial)	
406050031	Estudo eletrofisiológico terapêutico I (ablação de taquicardia atrial direita)	
406050058	Estudo eletrofisiológico terapêutico I (ablação do nódulo Archov-Tawara)	
**Ablação simples de circuitos em átrio esquerdo**		
406050139	Estudo eletrofisiológico terapêutico II (ablação de vias anômalas esquerdas)	2 introdutores (R$ 97), 1 agulha para punção transeptal (R$ 324) e 1 bainha para punção transeptal (R$ 324)
406050066	Estudo eletrofisiológico terapêutico II (ablação das vias anômalas múltiplas)	
406050104	Estudo eletrofisiológico terapêutico II (ablação de taquicardia atrial esquerda)	
**Ablação complexa de fibrilação atrial ou taquicardia atrial**		
406050090	Estudo eletrofisiológico terapêutico II (ablação de taquicardia atrial cicatricial)	2 introdutores (R$ 97), 1 agulha para punção transeptal (R$ 324) e 1 bainha para punção transeptal (R$ 324)
406050082	Estudo eletrofisiológico terapêutico II (ablação de taquicardia atrial cicatricial)	
406050074	Estudo eletrofisiológico terapêutico II (ablação de fibrilação atrial)	
**Ablação complexa de taquicardia ventricular**		
406050120	Estudo eletrofisiológico terapêutico II (ablação de taquicardia ventricular sustentada com cardiopatia estrutural)	2 introdutores (R$ 97)
406050112	Estudo eletrofisiológico terapêutico II (ablação de taquicardia ventricular idiopática do seio de Valsalva esquerdo)	

* Materiais compatíveis são recursos materiais utilizados nos estudos eletrofisiológicos terapêuticos.^10^

Analisou-se, também, o número de estabelecimentos habilitados em eletrofisiologia necessários por estado, considerando que o local deve ter pelo menos um estabelecimento a cada 2,6 milhões de habitantes, de acordo com o Ministério da Saúde.^11^

O custo foi analisado para cada grupo de procedimentos. As listas de materiais necessários para a ablação por radiofrequência foram definidas junto ao comitê e a base do valor foi o menor valor disponível no histórico de licitações realizadas pelos hospitais do SUS, extraído do portal ComprasNet.^12^ Além disso, estimou-se o valor atual de reembolso para cada código, que inclui serviços médicos e hospitalares, somado ao valor dos materiais compatíveis (Tabela S4).

A análise foi conduzida através do software Microsoft Office Excel^®^ (Microsoft Corporation, Redmond, WA, EUA). Os dados foram avaliados por meio de uma análise estatística descritiva com resultados expressos em frequência absoluta e relativa.

## Resultados

Em 2019, foram realizados 6.471 procedimentos de eletrofisiologia pelo SUS, sendo 53% dos procedimentos do grupo de estudo eletrofisiológico, 22% do grupo de ablação simples de circuitos em átrio direito, 18% do grupo de ablação simples de circuitos em átrio esquerdo e 8% dos grupos de ablação complexa.

Com relação ao perfil dos pacientes, 52% foram mulheres e a faixa etária mais frequente foi de 55 a 59 anos para ambos os sexos (Figura 1).

**Figura 1:**
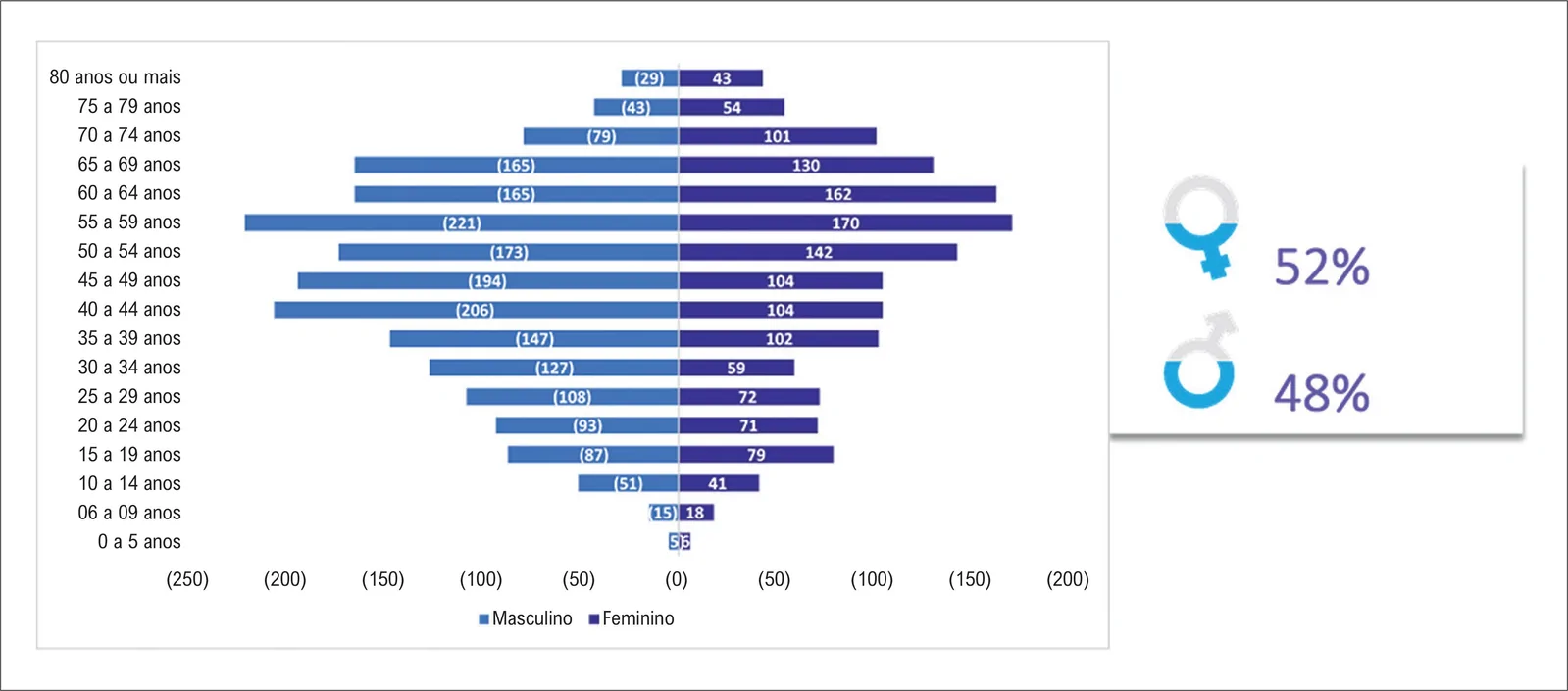
Quantidade de pacientes de acordo com sexo e faixa etária.

A Região Sul foi a que apresentou maior número de procedimentos realizados (3.112; 48%), seguida das regiões Sudeste (31%) e Centro-Oeste (10%). As regiões Norte e Nordeste apresentaram maior volume de habitantes por estabelecimentos habilitados, contabilizando 3.734.518 e 2.732.107 habitantes, respectivamente.

A Secretaria de Atenção à Saúde do Ministério da Saúde estabelece que o serviço de eletrofisiologia credenciado ao SUS deve realizar, no mínimo, 39 procedimentos por ano. A Tabela 2 e a Tabela 3 listam, respectivamente, os 15 estabelecimentos que mais realizaram procedimentos de todos os grupos e procedimentos complexos em 2019.

**Tabela 2:** Estabelecimentos com maior número de procedimentos em 2019

Estabelecimento	Município (UF)	Procedimentos	
		N	%
Hospital Angelina Caron	Campina Grande do Sul (PR)	742	11,5
Instituto de Cardiologia do Rio Grande do Sul	Porto Alegre (RS)	675	10,4
Instituto do Coração do HCFMUSP (INCOR)	São Paulo (SP)	422	6,5
Hospital São Vicente de Paulo	Passo Fundo (RS)	313	4,8
Instituto Dante Pazzanese de Cardiologia (IDPC)	São Paulo (SP)	311	4,8
Santa Casa de Misericórdia de Goiânia	Goiânia (GO)	252	3,9
Instituto de Cardiologia de Santa Catarina (ICSC)	São José (SC)	219	3,4
Hospital São Paulo da EPM/UNIFESP	São Paulo (SP)	199	3,1
Hospital de Caridade de Ijuí	Ijuí (RS)	143	2,2
Hospital de Messejana Dr. Carlos Alberto Studart Gomes (HM)	Fortaleza (CE)	141	2,2
Instituto de Cardiologia do Distrito Federal (ICDF)	Brasília (DF)	133	2,1
HCFMUSP de Ribeirão Preto	Ribeirão Preto (SP)	133	2,1
Hospital das Clínicas da UFG	Goiânia (GO)	129	2,0
Hospital Bruno Born	Lajeado (RS)	122	1,9
Santa Casa de Misericórdia de Juiz de Fora	Juiz de Fora (MG)	115	1,8

EPM/UNIFESP: Escola Paulista de Medicina da Universidade Federal de São Paulo; HCFMUSP: Hospital das Clínicas da Faculdade de Medicina da Universidade de São Paulo; UFG: Universidade Federal de Goiás.

**Tabela 3:** Estabelecimentos com maior número de procedimentos complexos em 2019

Estabelecimento	Município (UF)	Procedimentos	
		N	%
Hospital das Clínicas da UFG	Goiânia (GO)	52	10,0
Instituto do Coração do HCFMUSP (INCOR)	São Paulo (SP)	44	8,5
Hospital das Clínicas Samuel Libânio	Pouso Alegre (MG)	42	8,1
Hospital Angelina Caron	Campina Grande do Sul (PR)	37	7,1
Hospital Santa Casa de Vitória	Vitória (ES)	27	5,2
Hospital Universitário do Maranhão	São Luís (MA)	27	5,2
Instituto de Cardiologia de Santa Catarina (ICSC)	São José (SC)	25	4,8
Hospital das Clínicas da Universidade Federal Minas Gerais	Belo Horizonte (MG)	22	4,2
Hospital de Clínicas de Itajubá (HCI)	Itajubá (MG)	20	3,8
Santa Casa de Belo Horizonte	Belo Horizonte (MG)	17	3,3
Hospital São Vicente de Paulo	Passo Fundo (RS)	17	3,3
Instituto Dante Pazzanese de Cardiologia (IDPC)	São Paulo (SP)	17	3,3
Hospital Regional São Paulo	Xanxerê (SC)	16	3,1
HCFMUSP de Ribeirão Preto	Ribeirão Preto (SP)	15	2,9
Santa Casa de Misericórdia de Juiz de Fora	Juiz de Fora (MG)	15	2,9

EPM/UNIFESP: Escola Paulista de Medicina da Universidade Federal de São Paulo; HCFMUSP: Hospital das Clínicas da Faculdade de Medicina da Universidade de São Paulo; UFG: Universidade Federal de Goiás.

A Região Sudeste apresentou 37 estabelecimentos habilitados em eletrofisiologia e 29 realizaram procedimentos (78%), sendo que 12 realizaram pelo menos 39 procedimentos em 2019. Além disso, São Paulo e Rio de Janeiro não tinham o número necessário de estabelecimentos habilitados em eletrofisiologia por habitante.

A análise indicou 22 estabelecimentos habilitados na Região Sul, sendo que 21 realizaram algum procedimento (95%) e 15 realizaram pelo menos 39 procedimentos em 2019. Todos os estados da Região Sul apresentavam o número necessário de estabelecimentos habilitados em eletrofisiologia por habitante.

Com relação à Região Nordeste, a análise apontou que 21 estabelecimentos eram habilitados e 13 realizaram procedimentos (62%), enquanto 6 realizaram pelo menos 39 procedimentos em 2019. Ceará, Bahia, Paraíba, Maranhão e Piauí não apresentavam o número necessário de estabelecimentos habilitados em eletrofisiologia por habitante.

A Região Centro-Oeste contava com 7 estabelecimentos habilitados, sendo que 6 realizaram algum procedimento (86%) e 5 realizaram pelo menos 39 procedimentos em 2019. Todos os estados da Região Centro-Oeste apresentavam o número necessário de estabelecimentos habilitados em eletrofisiologia por habitante.

Por fim, a Região Norte tinha 5 estabelecimentos habilitados em eletrofisiologia e 3 que realizaram procedimentos em 2019 (60%), sendo que nenhum realizou pelo menos 39 procedimentos no ano. A Tabela 4 detalha o número de estabelecimentos habilitados por região e a proporção que realizou procedimentos em 2019.

**Tabela 4:** Número de estabelecimentos e proporção que realizou procedimentos por região no ano de 2019

Região	Estabelecimentos habilitados	Estabelecimentos que realizaram procedimentos	
		N	%
Sudeste	37	29	78
Sul	22	21	95
Centro-Oeste	7	5	86
Nordeste	21	13	62
Norte	5	3	60

Com base no número de estabelecimentos habilitados, as porcentagens de estabelecimentos que cumpriram o mínimo, de acordo com a portaria N° 384 de 26 de maio de 2006, foram: 32% no Sudeste, 68% no Sul, 29% no Nordeste e 71% no Centro-Oeste.^11^ No cenário nacional, 38 dos 92 estabelecimentos habilitados (41%) realizaram no mínimo os 39 procedimentos previstos na portaria. A distribuição regional dos centros habilitados para ablação no SUS em 2019 é apresentada na Figura Central.

### Disponibilidade de equipamentos necessários para o procedimento e deslocamento

Em relação à disponibilidade dos equipamentos necessários para a ablação, observou-se que, na Região Sudeste, 23 estabelecimentos possuíam todos esses equipamentos (62% dos 37 habilitados). O estado do Espírito Santo apresentou a maior taxa de deslocamento intermunicipal (84%), onde o paciente precisou se deslocar de seu município para realizar o procedimento.

Na Região Sul, todos os equipamentos necessários estavam presentes em 14 estabelecimentos (64% dos 22 habilitados) e o estado de Santa Catarina apresentou o maior deslocamento intermunicipal (85%). Na região Centro-Oeste, 2 estabelecimentos possuíam todos os equipamentos necessários (29% dos 7 habilitados) e o estado de Mato Grosso apresentou o maior deslocamento intermunicipal (85%). Na Região Nordeste, todos os equipamentos necessários estavam em 9 estabelecimentos (43% dos 21 habilitados), sendo que o estado de Pernambuco apresentou o maior deslocamento intermunicipal (77%). Na Região Norte, apenas 1 estabelecimento possuía os equipamentos necessários (20% dos 5 habilitados) e o maior deslocamento intermunicipal ocorreu no Pará, com 66%.

Desta forma, no panorama nacional, do ponto de vista de infraestrutura, 49 dos 92 (53%) estabelecimentos possuíam todos os equipamentos necessários para a realização de procedimentos de eletrofisiologia, e dentre os 38 estabelecimentos que realizaram pelo menos 39 procedimentos no ano de 2019, 30 possuíam todos os equipamentos (79%).

### Volume de procedimentos por grupo

A Tabela 5 apresenta o total de procedimentos por estabelecimentos realizados em 2019, sendo 8% de arritmias complexas. Procedimentos complexos foram realizados em 44 estabelecimentos habilitados (48%), enquanto os demais procedimentos foram realizados em 72 dos 92.

**Tabela 5:** Volume de procedimentos realizados por grupo e proporção de estabelecimentos que realizaram

Grupo	Procedimentos (%)	Estabelecimentos (%)
Ablação complexa de taquicardia ventricular	279 (4)	39 (42)
Ablação complexa de fibrilação atrial ou taquicardia atrial	241 (4)	33 (36)
Ablação simples de circuitos em átrio esquerdo	1.421 (22)	58 (63)
Ablação simples de circuitos em átrio direito	3.366 (53)	68 (74)
Estudo eletrofisiológico	1.164 (18)	59 (64)

### Custo por grupo de procedimento

O custo total dos procedimentos e os valores reembolsados pelo SUS foram dispostos na Tabela S4 e sumarizados na Tabela 6. Para o grupo ablação complexa de taquicardia ventricular, o valor de reembolso foi composto por R$ 4.195 do código SIGTAP 04.06.05.011-2 (R$ 3.328 de serviço hospitalar e R$ 867 de serviço profissional) e 2 introdutores (R$ 97). O custo apenas dos materiais (sem considerar outras despesas hospitalares e honorários profissionais) foi 4,8 vezes maior que o valor reembolsado.

**Tabela 6:** Custo total de procedimentos por grupo

Grupo	Custo dos materiais por procedimento (R$)	Valor reembolsado por procedimento (R$)
Ablação complexa de taquicardia ventricular	20.000	4.389
Ablação complexa de fibrilação atrial ou taquicardia atrial	22.300	6.251
Ablação simples de circuitos em átrio esquerdo	7.000	5.792
Ablação simples de circuitos em átrio direito	6.400	4.576
Estudo eletrofisiológico	3.800	2.766

No grupo ablação complexa de fibrilação atrial ou taquicardia atrial, o valor de reembolso incluiu R$ 5.409 do código SIGTAP 04.06.05.009-0 (R$ 4.542 de serviço hospitalar e R$ 867 de serviço profissional) e 2 introdutores (R$ 97), 1 agulha para punção transeptal (R$ 324) e 1 bainha para punção transeptal (R$ 324). Nesse grupo, o custo dos materiais foi 4,1 vezes maior que o valor reembolsado pelo SUS.

O valor de reembolso no SUS do grupo ablação simples de circuitos em átrio esquerdo foi constituído por R$ 4.950 do código SIGTAP 04.06.05.013-9 (R$ 4.084 de serviço hospitalar e R$ 867 de serviço profissional) e 2 introdutores (R$ 97), 1 agulha para punção transeptal (R$ 324) e 1 bainha para punção transeptal (R$ 324). O custo com materiais foi 1,4 vezes maior que o valor reembolsado.

Para o grupo ablação simples de circuitos em átrio direito, o valor de reembolso foi composto por R$ 4.382 do código SIGTAP 04.06.05.003-1 (R$ 3.515 de serviço hospitalar e R$ 867 de serviço profissional) e 2 introdutores (R$ 97).

Por fim, para o grupo estudo eletrofisiológico, o valor total de reembolso foi de R$ 2.572 do código SIGTAP 04.06.05.001-5 e 2 introdutores (R$ 97). Tanto no grupo 2 quanto no grupo 1, os custos dos materiais foram 1,5 vezes maiores que os valores reembolsados pelo SUS.

## Discussão

Em 2019, foram realizados 6.471 procedimentos de eletrofisiologia pelo SUS no Brasil. Se levarmos em consideração os dados do estudo RECALL,^13^ que mostrou que 5 a cada 100 pacientes com fibrilação atrial são submetidos à ablação e do *Global Burden of Disease* 2021^14^ que estima que a cada ano 170.000 novos casos de fibrilação atrial ocorrem no país, espera-se que, cerca de 8.500 pacientes sejam submetidos anualmente à ablação de fibrilação atrial no Brasil. Só esse número já é superior ao número total de ablações realizadas no Brasil, considerando-se todos os tipos de arritmias.

No presente estudo, identificou-se que a Região Sul foi a que apresentou maior número de procedimentos realizados (48%), seguida das regiões Sudeste (31%) e Centro-Oeste (10%); este resultado não está de acordo com as maiores concentrações populacionais do Brasil, que são encontradas nas regiões Sudeste (41,8%), Nordeste (26,9%) e Sul (14,7%), nessa ordem. As regiões Norte e Centro-Oeste representam 8,5% e 8% da população, respectivamente.

No panorama nacional, do ponto de vista de infraestrutura, 49 dos 92 (53%) estabelecimentos do país possuíam todos os equipamentos necessários para a realização de ablação de arritmias cardíacas, o que denota escassez de centros totalmente equipados para o procedimento. Além disso, a realização do procedimento exige profissionais experientes, que ainda apresentam disponibilidade limitada em países de média e baixa renda.^15,16^ Embora a formação especializada seja oferecida em grande parte por instituições públicas, o que reduz a necessidade de contratação externa, há dificuldade em manter esses especialistas no sistema público devido às limitações estruturais, falta de remuneração compatível com a complexidade do trabalho e pouca estabilidade profissional.^17,18^ Os estabelecimentos habilitados para realização de procedimentos eletrofisiológicos estão concentrados nas regiões Sul e Sudeste mesmo havendo infraestrutura e centros habilitados em todas as regiões. As outras regiões apresentam um número muito pequeno de estabelecimentos que concentram a realização dos procedimentos. Nesse sentido, a presença de estabelecimentos com disponibilidade de profissionais e equipamentos adequados são indispensáveis para realização segura de procedimentos de ablações para arritmias e sustentabilidade desses serviços.

As regiões Norte e Nordeste apresentaram, neste estudo, menor número de estabelecimentos habilitados por habitante. Destaca-se que a Região Norte está localizada em grande parte na Amazônia, uma região caracterizada por uma floresta densa com grandes rios e alta biodiversidade, com cidades escassamente povoadas. Várias comunidades estão localizadas nas margens dos rios. O transporte para essas comunidades ocorre principalmente por barcos e depende das condições climáticas. Essas características prejudicam o acesso adequado à assistência médica na região, um problema também agravado pelo menor nível socioeconômico do Norte do Brasil em comparação com outras regiões brasileiras.^15^

Em relação à Região Nordeste, um estudo analisou as hospitalizações e óbitos por transtornos de condução e arritmias na Bahia. Os dados mostraram que o estado liderou a Região Nordeste com 15.249 hospitalizações, correspondendo a 32,1% do total, e ocupou a segunda posição em óbitos por arritmias, com 989 (25,8%).^19^ Na Bahia, as arritmias cardíacas representam uma parte considerável das hospitalizações e óbitos tanto em hospitais públicos quanto em privados conveniados ao SUS.^19^ Esses dados demandam atenção especial das autoridades de saúde para diminuir a morbidade e mortalidade associadas a essas condições. Apesar dos avanços no tratamento das arritmias, os óbitos associados continuam a representar uma complicação significativa.^19^

É importante destacar a questão dos custos dos materiais *versus* os valores reembolsados. Esse estudo mostrou que os custos dos materiais (sem considerar outros custos hospitalares e honorários profissionais) são 1,5 a 4,8 vezes maiores que os valores reembolsados. Nesse contexto, uma análise de impacto orçamentário com dados brasileiros mostrou que, à medida que o valor do procedimento excede o reembolso realizado pelo SUS, os custos totais, incluindo serviços hospitalares e honorários médicos, devem ter o financiamento complementado.^17^ Essa discrepância pode provocar escassez de materiais e limitar o número de procedimentos realizados. A ablação para o tratamento de arritmias é um procedimento complexo, pois requer técnica especializada e procedimento eletrofisiológico avançado realizado em centro de referência, sendo necessária remuneração adequada para garantir a viabilidade do serviço no SUS.^20^ Esse fato pode deixar uma parcela da população desassistida e dependente de tratamento medicamentoso, mesmo que os medicamentos tenham menor eficácia no controle das arritmias quando comparado ao tratamento ablativo. Além disso, os eventos adversos, a baixa adesão ao tratamento e as taxas de desistência são maiores com fármacos.^21–25^

Os distúrbios do ritmo cardíaco afetam milhões de pessoas em todo o mundo e são responsáveis por altas taxas de hospitalizações e de procedimentos diagnósticos e terapêuticos.^26^ O envelhecimento global da população contribui para o aumento da prevalência de arritmias, acarretando peso significativo sobre os sistemas de saúde e profissionais responsáveis por essas populações.^27,28^ Esse estudo mostrou que para manter o acesso ao tratamento adequado das arritmias no Brasil é necessário aumentar o número de centros adequadamente equipados, reduzir a necessidade de grandes deslocamentos pelos pacientes e realizar ajuste no valor de reembolso do custo do procedimento.

### Limitações

A precisão dos dados obtidos do DATASUS (SIH/SUS) e do CNES não foi verificada, partindo-se do princípio de que o seu preenchimento seguiu as recomendações estabelecidas e que cada centro executor auditou e validou corretamente as informações alimentadas por eles. Em decorrência da utilização de dados secundários, este estudo pode apresentar limitações associadas a subnotificação de procedimentos, inconsistências na codificação dos registros hospitalares e heterogeneidade regional na qualidade das informações. Além disso, a discrepância observada entre o custo real gasto e o valor reembolsado reforça a hipótese de subestimação relacionada às limitações da base de dados ou do método de coleta. Além disso, a análise se restringiu ao ano de 2019, último ano pré-pandemia de COVID-19. A escolha se justifica pela variação excepcional no número de internações observadas no período pandêmico, o que poderia enviesar os resultados da análise. No entanto, a escolha deste ponto temporal traz limitações relativas à generalização dos resultados, uma vez que, em 2022, os procedimentos eletrofisiológicos tiveram reajuste do seu valor de reembolso. Assim, as diferenças observadas neste estudo podem ser maiores do que as observadas atualmente.

### Importância do estudo

O Ministério da Saúde/Secretaria de Ciência, Tecnologia e Inovação e do Complexo Econômico-Industrial da Saúde, por meio da portaria SECTICS/MS N° 35 de 27 maio de 2025, publicada no Diário Oficial da União em 29 de maio de 2025, tornou pública a decisão de incorporar, no âmbito do SUS, o cateter com força de contato e o mapeamento eletroanatômico tridimensional para ablação por radiofrequência no tratamento de pacientes adultos com arritmias cardíacas complexas.

## Conclusões

No âmbito do SUS, existe disparidade regional significativa na disponibilização de serviços de eletrofisiologia, especialmente nas regiões Norte e Nordeste. A insuficiência de centros equipados, o baixo desempenho de diversos centros que não realizam número mínimo de procedimentos preconizados e o baixo reembolso dos procedimentos são desafios críticos que precisam ser superados para melhorar o acesso ao tratamento de arritmias cardíacas no Brasil.

## Data Availability

Os conteúdos subjacentes ao texto da pesquisa estão contidos no manuscrito.

## Material suplementar

Material suplementar Para informação adicional, por favor, clique aqui.
