# Supplementary material for: Ablação por Radiofrequência para Tratamento de Arritmias Cardíacas no Brasil: O Cenário do Sistema Único de Saúde
Source: Arq Bras Cardiol. 2026 May 21;123(4):e20250411. [Article in Portuguese] doi: 10.36660/abc.20250411 (PMC13712640; doi:10.36660/abc.20250411)
Supplement: Supplementary file 1 [file 0066-782x-abc-123-4-e20250411-Supp01-en.pdf]

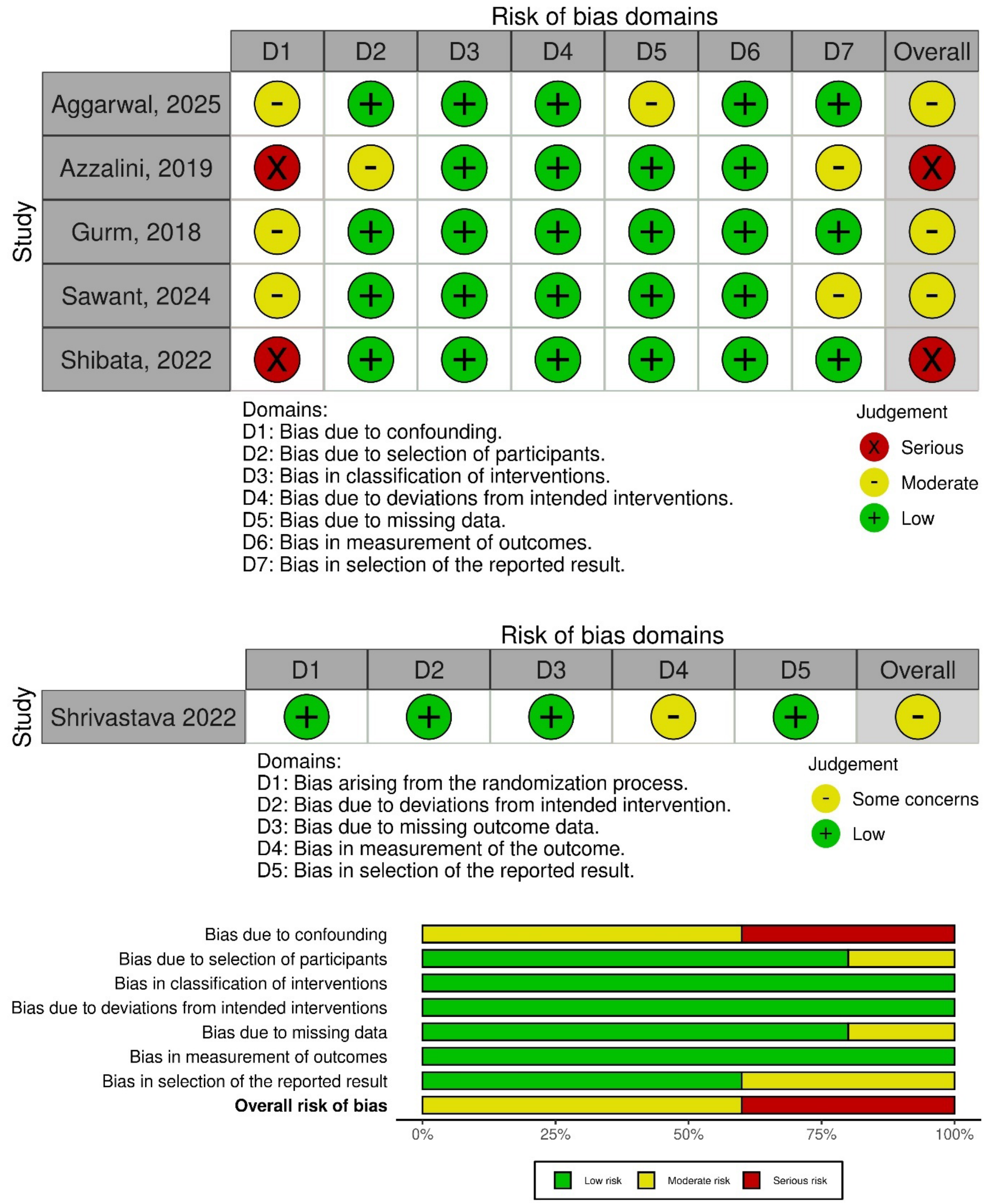

SUPPLEMENTAL FIGURE 1 | Upper and central panels: Risk of bias assessment for included studies, according to ROBINS-I and RoB 2 tools across seven domains. Green (+) indicates low, yellow (−), moderate, and red (X), serious risk of bias. Lower panel: Risk of bias summary across seven domains, showing proportions of low (green), moderate (yellow), and serious (red) risk of bias, according to ROBINS-I and RoB 2 tools.

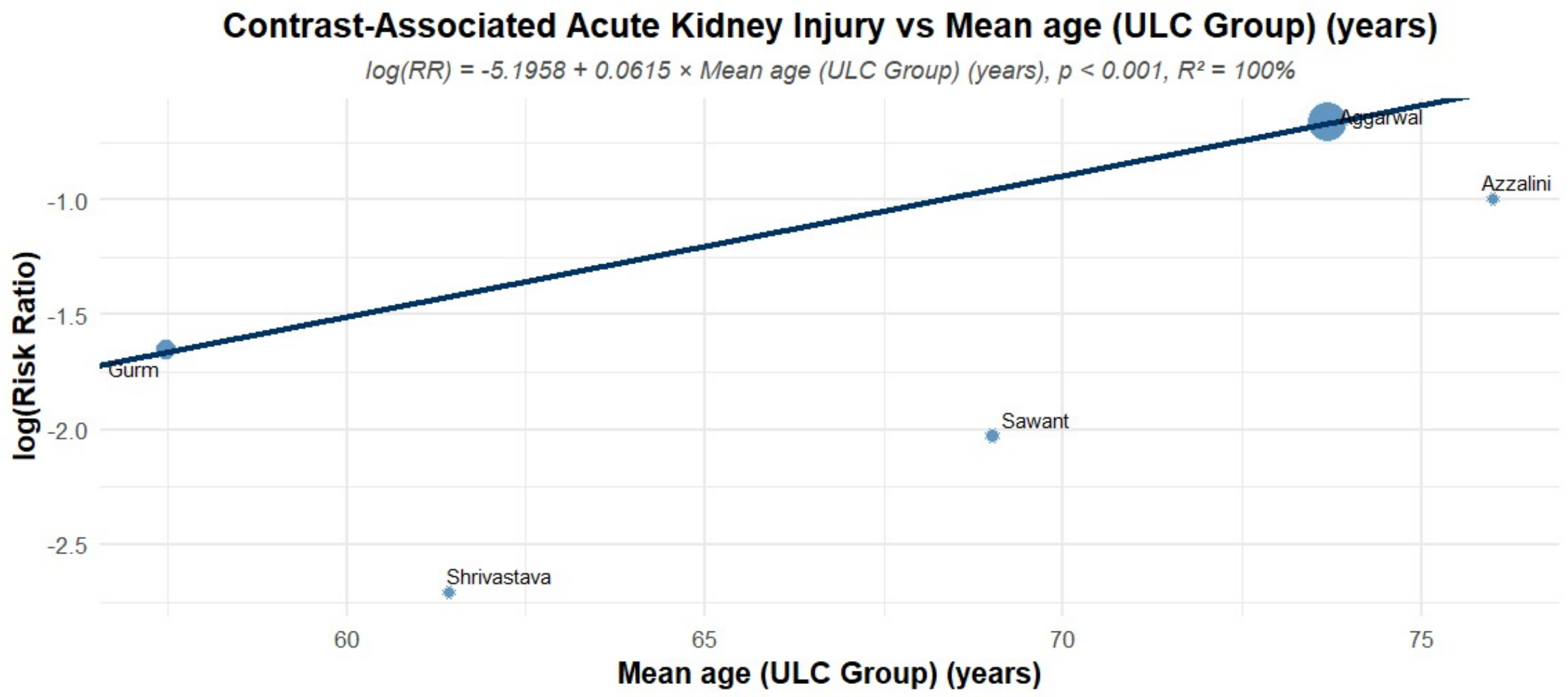

SUPPLEMENTAL FIGURE 2 | Meta-regression analysis: Risk of Contrast-Associated Acute Kidney Injury versus Mean Age for Ultra-Low Contrast Percutaneous Coronary Intervention.

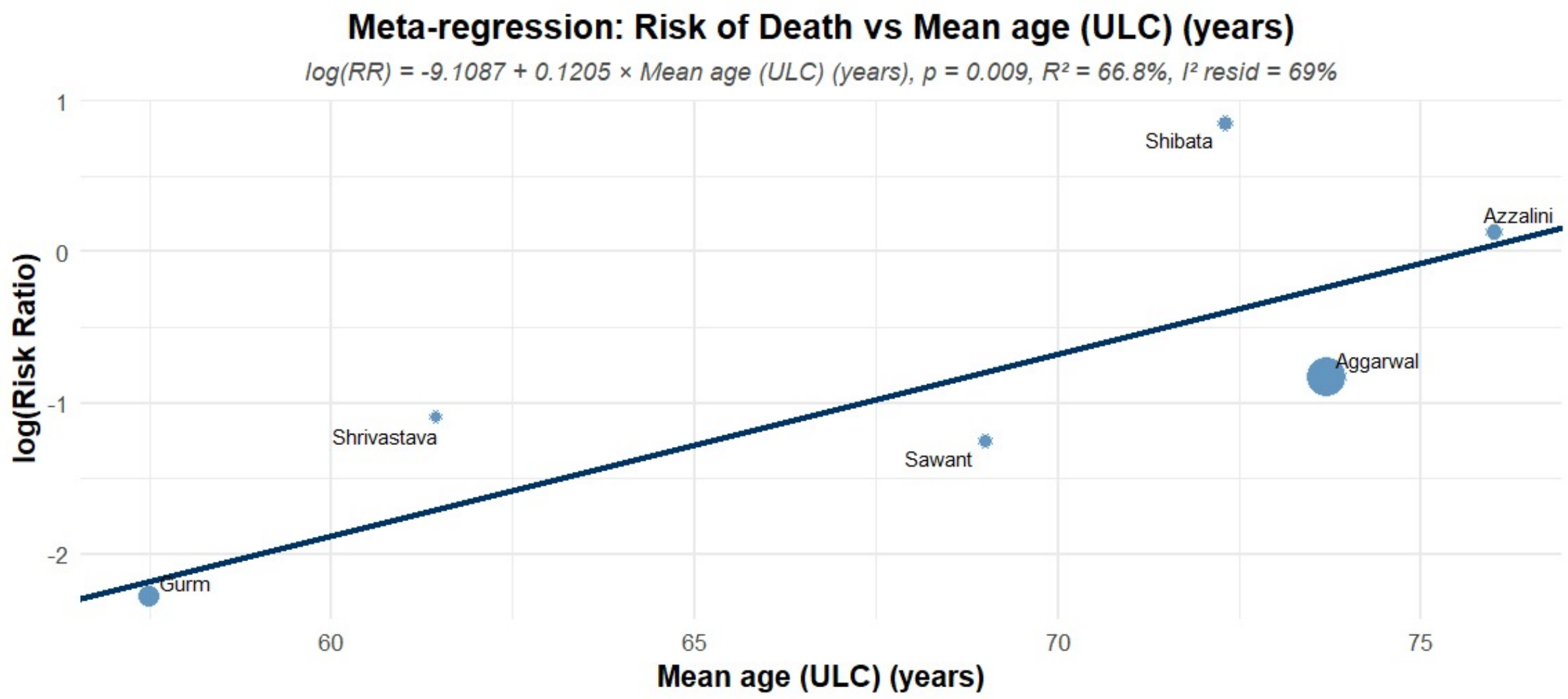

SUPPLEMENTAL FIGURE 3 | Meta-regression analysis: Risk of Death versus Mean Age for Ultra-Low Contrast Percutaneous Coronary Intervention.
