# Supplementary material for: Ablação por Radiofrequência para Tratamento de Arritmias Cardíacas no Brasil: O Cenário do Sistema Único de Saúde
Source: Arq Bras Cardiol. 2026 May 21;123(4):e20250411. [Article in Portuguese] doi: 10.36660/abc.20250411 (PMC13712640; doi:10.36660/abc.20250411)
Supplement: Translation

# Translation: Radiofrequency Ablation for Treating Cardiac Arrhythmias in Brazil: Status in the Unified Health System

**DOI:** 10.36660/abc.20250411i

**Authors:** Ricardo Alkmim Teixeira, Elerson Arfelli, Fatima Dumas Cintra, Jose Carlos Moura Jorge, Alexsandro Alves Fagundes, Martino Martinelli

**Affiliations:** 1 Pouso Alegre MG Brazil Universidade do Vale do Sapucaí – UNIVAS, Pouso Alegre, MG – Brazil; 2 Ribeirão Preto SP Brazil Universidade de São Paulo Hospital das Clínicas da Faculdade de Medicina de Ribeirão Preto, Ribeirão Preto, SP – Brazil; 3 São Paulo SP Brazil Universidade Federal de São Paulo, São Paulo, SP – Brazil; 4 Curitiba PR Brazil Irmandade da Santa Casa de Misericórdia de Curitiba, Curitiba, PR – Brazil; 5 Curitiba PR Brazil Pontifícia Universidade Católica do Paraná, Curitiba, PR – Brazil; 6 Salvador BA Brazil Universidade do Estado da Bahia, Salvador, BA – Brazil; 7 São Paulo SP Brazil Instituto do Coração do Hospital das Clínicas da Faculdade de Medicina da Universidade de São Paulo, São Paulo, SP – Brazil

**Keywords:** Cardiac Arrhythmias, Radiofrequency Ablation, Barriers to Access of Health Services, Unified Health System

## Abstract

**Background:**

Cardiac arrhythmias compromise quality of life and are associated with greater morbidity and mortality. Pharmacological treatment generally has limited efficacy and often causes adverse effects. Radiofrequency ablation is effective and indicated for treating various arrhythmias; however, this therapeutic modality is not widely available in Brazil through the Unified Health System (SUS).

**Objective:**

To analyze ablation procedures in the treatment of cardiac arrhythmias performed in Brazil by the SUS in 2019, in order to identify factors that limit the availability of this treatment to patients served by the SUS.

**Methods:**

This retrospective study analyzed data from the Hospital Information System (SIH/SUS) and the National Registry of Health Facilities (CNES). The procedures were classified according to their complexity. The infrastructure of the accredited centers was assessed based on the availability of necessary equipment and trained professionals.

**Results:**

In 2019, 6,471 electrophysiology procedures were performed by the SUS in Brazil, and 82% were low or medium complexity (groups 1 to 3). The South and Southeast Regions concentrated the majority of procedures (48% and 31%, respectively). Only 53% of centers had registered all the necessary equipment. The cost of materials exceeded the amount reimbursed by the SUS by 1.5- to 4.8-fold.

**Conclusions:**

Within the SUS, there is a significant regional disparity in the availability of electrophysiology services, especially in the North and Northeast Regions. The lack of properly equipped centers and the low reimbursement of procedures are critical challenges to improving access to arrhythmia treatment in Brazil.

## Introduction

Brazil's Unified Health System (SUS) is one of the largest public health systems worldwide, and it offers universal coverage to the entire Brazilian population. In 2019, the SUS served as the exclusive health system for 76% of the Brazilian population, which has been estimated at 210 million people.^1^

According to Cardiovascular Statistics – Brazil 2020, by the Brazilian Society of Cardiology, cardiovascular diseases generate the highest hospitalization costs for the SUS and are the leading cause of pensions granted for disability; they are also the leading cause of morbidity. In this context, atrial fibrillation, the most frequent sustained cardiac arrhythmia, caused 321,866 hospitalizations (14.7 per 100,000 inhabitants) within the SUS, between 2008 and 2018,^1^ and was responsible for approximately BRL 3.9 billion of costs in 2015.^2^

Technological advances, combined with a more detailed understanding of the pathophysiological mechanisms of arrhythmias, have enabled the development of more precise diagnostic methods and effective treatments.^3^ In this context, catheter ablation seeks to eliminate small areas of abnormal cardiac tissue that cause electrical disturbances in the heart. This technique is effective in treating various forms of arrhythmias, reducing the need for continuous medication and improving patient quality of life.^3^

As Brazil is a developing, continent-sized country with major socioeconomic disparities, there are many gaps and regional differences in medical care, including access to the best strategies for treating cardiac arrhythmias, especially for patients who depend on the SUS. It is worth underscoring that the SUS does not currently provide reimbursement for the treatment of complex arrhythmias (atrial fibrillation, scar-related atrial tachycardia, and ventricular arrhythmias) by means of the current reference technique (electroanatomical mapping and contact force catheter). In this context, the National Commission for the Incorporation of Technologies in the SUS (CONITEC), motivated by a request from the Brazilian Society of Cardiac Arrhythmias (SOBRAC), recently issued a favorable opinion on the incorporation of electroanatomical mapping and contact force catheters for the ablation of complex arrhythmias by the SUS.^4^ Nonetheless, there is a lack of structured data on the use of modern techniques and clinical results from the perspective of Brazilian public health.^5^

Accordingly, the objective of this study was to analyze the status of cardiac arrhythmia treatment within the SUS in Brazil, based on the year 2019, due to the COVID-19 pandemic, which continued until early 2023.

## Methods

This retrospective study was conducted with a quantitative approach using databases from the Information Technology Department of the Unified Health System (DATASUS), composed of information from across Brazil reported by official documents, such as hospitalization authorizations, medication dispensing paperwork, and death and birth records.

Two systems were analyzed: the subset database of the DATASUS Hospital Information System (SIH/SUS), supported by Hospital Information Authorization (AIH), and the database of the National Registry of Health Facilities (CNES), the official information system for registering information on all health establishments in Brazil.^6^ The data extracted from the database were used in their original form; no missing data were added or data were deleted.

The AIH is generated at the time of hospitalization in a public or private provider affiliated with the SUS and sent to the manager of the service provider unit. On a monthly basis, managers send a magnetic file with data from all hospitalizations that occurred in Brazil to the Ministry of Health for the billing of hospital services provided within the SUS.^6^

Data from the SIH/SUS database were extracted with filters related to diagnosis, according to the Tenth Revision of the International Classification of Diseases (Table S1), and the procedure performed (Table S2).

Analysis of the CNES database was carried out by selecting the accreditations 801 – High Complexity Cardiovascular Care Unit, 802 – High Complexity Cardiovascular Reference Center, and 807 – Electrophysiology Laboratory; the latter required at least one of the two former (801 or 802). Cardiologists working at the analyzed centers from the CNES database should be listed in the roster of qualified professionals in the field of electrophysiology, available on the SOBRAC website (www.sobrac.org).^7,8^

The necessary equipment for the procedures was defined in collaboration with the SOBRAC committee and in accordance with Ordinance Number 210 of June 15, 2004, of the Secretariat of Health Care.^9^Table S3 lists the necessary equipment. Polygraph, 3-dimensional mapping, and radiofrequency generator were not included, as they are portable and can be brought to the facility on the day of the procedure by the material suppliers.

For analysis, procedures were divided into groups according to their complexity (Table 1).

**Table 1:** Procedure groups used in analyses

Electrophysiological study		Compatible materials*
406050015	Diagnostic electrophysiological study	2 introducers (BRL 97)
**Simple ablation of right atrial pathways**		
406050040	Therapeutic electrophysiological study I	2 introducers (BRL 97)
406050023	Therapeutic electrophysiological study I (ablation of atrial flutter)	
406050031	Therapeutic electrophysiological study I (ablation of right atrial tachycardia)	
406050058	Therapeutic electrophysiological study I (ablation of the atrioventricular node)	
**Simple ablation of left atrial pathways**		
406050139	Therapeutic electrophysiological study II (ablation of anomalous left pathways)	2 introducers (BRL 97), 1 transseptal puncture needle (BRL 324), and 1 transseptal puncture sheath (BRL 324)
406050066	Therapeutic electrophysiological study II (ablation of multiple anomalous pathways)	
406050104	Therapeutic electrophysiological study II (ablation of left atrial tachycardia)	
**Complex ablation of atrial fibrillation or atrial tachycardia**		
406050090	Therapeutic electrophysiological study II (ablation of scar-related atrial tachycardia)	2 introducers (BRL 97), 1 transseptal puncture needle (BRL 324), and 1 transseptal puncture sheath (BRL 324)
406050082	Therapeutic electrophysiological study II (ablation of scar-related atrial tachycardia)	
406050074	Therapeutic electrophysiological study II (ablation of atrial fibrillation)	
**Complex ablation of ventricular tachycardia**		
406050120	Therapeutic electrophysiological study II (ablation of sustained ventricular tachycardia with structural heart disease)	2 introducers (BRL 97)
406050112	Therapeutic electrophysiological study II (ablation of idiopathic ventricular tachycardia of the left Valsalva sinus)	

* Compatible materials indicate material resources used in therapeutic electrophysiological studies.^10^

The number of accredited electrophysiology centers required per state was also analyzed, considering that a location should have at least one center per 2.6 million inhabitants, according to the Brazilian Ministry of Health.^11^

Costs were analyzed for each group of procedures. The lists of materials required for radiofrequency ablation were defined in collaboration with the committee, and the basis for the value was the lowest value available in the history of bids carried out by SUS hospitals, extracted from the ComprasNet portal.^12^ The current reimbursement value for each code was also estimated, including medical and hospital services, plus the value of compatible materials (Table S4).

Analyses were carried out using Microsoft Office Excel^®^ (Microsoft Corporation, Redmond, WA, USA). Data were assessed by means of descriptive statistical analysis, with results expressed as absolute and relative frequency.

## Results

In 2019, 6,471 electrophysiology procedures were performed by the SUS, 53% in the electrophysiological study group, 22% in the simple ablation of right atrial pathways group, 18% in the simple ablation of left atrial pathways group, and 8% in the complex ablation groups.

In relation to patient profile, 52% were women, and the most frequent age range was 55 to 59 years for both sexes (Figure 1).

**Figure 1:** Distribution of patients by sex and age range.

The South Region had the highest number of procedures performed (3,112; 48%), followed by the Southeast (31%) and Central-West (10%). The North and Northeast Regions had the highest number of inhabitants per accredited center, totaling 3,734,518 and 2,732,107 inhabitants, respectively.

The Secretariat of Health Care of the Brazilian Ministry of Health has established that SUS-accredited electrophysiology centers must perform at least 39 procedures per year. Table 2 lists the 15 centers that performed the highest number of procedures from all groups, and Table 3 lists the 15 centers that performed the highest number of complex procedures in 2019.

**Table 2:** Centers that performed the highest number of procedures in 2019

Center	City, State	Procedures	
		N	%
Hospital Angelina Caron	Campina Grande do Sul (PR)	742	11.5
Instituto de Cardiologia do Rio Grande do Sul	Porto Alegre (RS)	675	10.4
Instituto do Coração do HCFMUSP (INCOR)	São Paulo (SP)	422	6.5
Hospital São Vicente de Paulo	Passo Fundo (RS)	313	4.8
Instituto Dante Pazzanese de Cardiologia (IDPC)	São Paulo (SP)	311	4.8
Santa Casa de Misericórdia de Goiânia	Goiânia (GO)	252	3.9
Instituto de Cardiologia de Santa Catarina (ICSC)	São José (SC)	219	3.4
Hospital São Paulo da EPM/UNIFESP	São Paulo (SP)	199	3.1
Hospital de Caridade de Ijuí	Ijuí (RS)	143	2.2
Hospital de Messejana Dr. Carlos Alberto Studart Gomes (HM)	Fortaleza (CE)	141	2.2
Instituto de Cardiologia do Distrito Federal (ICDF)	Brasília (DF)	133	2.1
HCFMUSP de Ribeirão Preto	Ribeirão Preto (SP)	133	2.1
Hospital das Clínicas da UFG	Goiânia (GO)	129	2.0
Hospital Bruno Born	Lajeado (RS)	122	1.9
Santa Casa de Misericórdia de Juiz de Fora	Juiz de Fora (MG)	115	1.8

EPM/UNIFESP: Escola Paulista de Medicina da Universidade Federal de São Paulo; HCFMUSP: Hospital das Clínicas da Faculdade de Medicina da Universidade de São Paulo; UFG: Universidade Federal de Goiás.

**Table 3:** Centers that performed the highest number of complex procedures in 2019

Center	City, State	Procedures	
		N	%
Hospital das Clínicas da UFG	Goiânia (GO)	52	10.0
Instituto do Coração do HCFMUSP (INCOR)	São Paulo (SP)	44	8.5
Hospital das Clínicas Samuel Libânio	Pouso Alegre (MG)	42	8.1
Hospital Angelina Caron	Campina Grande do Sul (PR)	37	7.1
Hospital Santa Casa de Vitória	Vitória (ES)	27	5.2
Hospital Universitário do Maranhão	São Luís (MA)	27	5.2
Instituto de Cardiologia de Santa Catarina (ICSC)	São José (SC)	25	4.8
Hospital das Clínicas da Universidade Federal Minas Gerais	Belo Horizonte (MG)	22	4.2
Hospital de Clínicas de Itajubá (HCI)	Itajubá (MG)	20	3.8
Santa Casa de Belo Horizonte	Belo Horizonte (MG)	17	3.3
Hospital São Vicente de Paulo	Passo Fundo (RS)	17	3.3
Instituto Dante Pazzanese de Cardiologia (IDPC)	São Paulo (SP)	17	3.3
Hospital Regional São Paulo	Xanxerê (SC)	16	3.1
HCFMUSP de Ribeirão Preto	Ribeirão Preto (SP)	15	2.9
Santa Casa de Misericórdia de Juiz de Fora	Juiz de Fora (MG)	15	2.9

EPM/UNIFESP: Escola Paulista de Medicina da Universidade Federal de São Paulo; HCFMUSP: Hospital das Clínicas da Faculdade de Medicina da Universidade de São Paulo; UFG: Universidade Federal de Goiás.

### Number of centers and procedure volume

In the Southeast Region, there were 37 accredited electrophysiology centers; 29 of them performed at least 1 procedure (78%), and 12 performed at least 39 procedures in 2019. Moreover, São Paulo and Rio de Janeiro did not meet the recommended number of centers per inhabitant.

The analysis identified 22 accredited centers in the South Region; 21 of them performed at least 1 procedure (95%), and 15 performed at least 39 procedures in 2019. All states in the South Region met the recommended number of centers per inhabitant.

Regarding the Northeast Region, the analysis indicated that 21 centers were accredited; 13 of them performed at least 1 procedure (62%), and 6 performed at least 39 procedures in 2019. The states of Ceará, Bahia, Paraíba, Maranhão, and Piauí did not meet the recommended number of centers per inhabitant.

The Central-West Region had 7 accredited centers; 6 of them performed at least 1 procedure (86%), and 5 performed at least 39 procedures in 2019. All states in the Central-West Region met the recommended number of centers per inhabitant.

Finally, the North Region had 5 accredited electrophysiology centers; 3 of them performed at least 1 procedure (60%), and none performed 39 procedures in 2019. Table 4 details the number of accredited centers by region and the proportion that performed procedures in 2019.

**Table 4:** Number of accredited centers and the proportion that performed at least 1 procedure by region in 2019

Region	Accredited centers	Centers that performed procedures (%)	
		N	%
Southeast	37	29	78
South	22	21	95
Central-West	7	5	86
Northeast	21	13	62
North	5	3	60

Based on the number of accredited centers, the proportion that met the minimum requirements stipulated by Ordinance Number 384 of May 26, 2006, was as follows, by region: 32% in the Southeast, 68% in the South, 29% in the Northeast, and 71% in the Central-West.^11^ On the national level, 38 of the 92 accredited centers (41%) performed at least 39 procedures, as stipulated by the ordinance. The Central Illustration displays the regional distribution of centers accredited to perform ablation within the SUS in 2019.

### Availability of necessary equipment for the procedure and patient transportation

Regarding the availability of equipment needed for ablation, in the Southeast Region, 23 accredited centers had all of the equipment (62% of 37). The state of Espírito Santo demonstrated the highest rate of intermunicipal patient travel (84%), where patients needed to travel from their home municipality to undergo a procedure.

In the South Region, all the necessary equipment was present in 14 centers (64% of 22), and the state of Santa Catarina demonstrated the highest rate of intermunicipal patient travel (85%). In the Central-West region, 2 centers had all the necessary equipment (29% of 7), and the state of Mato Grosso demonstrated the highest rate of intermunicipal patient travel (85%). In the Northeast Region, all the necessary equipment was present in 9 centers (43% of 21), and the state of Pernambuco demonstrated the highest rate of intermunicipal patient travel (77%). In the Northern Region, only 1 center had the necessary equipment (20% of 5), and Pará had the highest rate of intermunicipal patient travel (66%).

Nationwide, regarding infrastructure, 49 of the 92 (53%) centers had all the equipment necessary to perform electrophysiology procedures; among the 38 centers that performed at least 39 procedures in 2019, 30 had all the necessary equipment (79%).

### Procedure volume by group

Table 5 displays the total number of procedures performed by centers in 2019; 8% of all procedures were in complex arrhythmias. Complex procedures were performed in 44 accredited centers (48%), and the remaining procedures were performed in 72 of the 92 centers.

**Table 5:** Volume of procedures performed by group and proportion of establishments that performed them

Group	Procedures (%)	Centers (%)
Complex ablation of ventricular tachycardia	279 (4)	39 (42)
Complex ablation of atrial fibrillation or atrial tachycardia	241 (4)	33 (36)
Simple ablation of left atrial pathways	1,421 (22)	58 (63)
Simple ablation of right atrial pathways	3,366 (53)	68 (74)
Electrophysiological study	1,164 (18)	59 (64)

### Cost per procedure group

The total cost of the procedures and the amounts reimbursed by the SUS are presented in Table S4 and summarized in Table 6. For the complex ablation of ventricular tachycardia group, the reimbursement amount consisted of BRL 4,195 from SIGTAP code 04.06.05.011-2 (BRL 3,328 for hospital services and BRL 867 for professional services), in addition to 2 introducers (BRL 97). The cost of materials alone (without considering other hospital expenses and professional fees) was 4.8 times greater than the reimbursed amount.

**Table 6:** Total costs of procedures by group

Group	Cost of materials per procedure (BRL)	Value reimbursed per procedure (BRL)
Complex ablation of ventricular tachycardia	20,000	4,389
Complex ablation of atrial fibrillation or atrial tachycardia	22,300	6,251
Simple ablation of left atrial pathways	7,000	5,792
Simple ablation of right atrial pathways	6,400	4,576
Electrophysiological study	3,800	2,766

In the complex ablation of atrial fibrillation or atrial tachycardia group, the reimbursement amount included BRL 5,409 from SIGTAP code 04.06.05.009-0 (BRL 4,542 for hospital services and BRL 867 for professional services), in addition to 2 introducers (BRL 97), 1 needle for transseptal puncture (BRL 324), and 1 sheath for transseptal puncture (BRL 324). In this group, the cost of materials was 4.1 times greater than the amount reimbursed by the SUS.

The reimbursement amount in the SUS for the simple ablation of left atrial pathways group consisted of BRL 4,950 from SIGTAP code 04.06.05.013-9 (BRL 4,084 for hospital services and BRL 867 for professional services), in addition to 2 introducers (BRL 97), 1 needle for transseptal puncture (BRL 324), and 1 sheath for transseptal puncture (BRL 324). The cost of materials was 1.4 times greater than the amount reimbursed by the SUS.

For the simple ablation of right atrial pathways group, the reimbursement amount consisted of BRL 4,382 from SIGTAP code 04.06.05.003-1 (BRL 3,515 for hospital services and BRL 867 for professional services), in addition to 2 introducers (BRL 97).

Finally, for the electrophysiological study group, the total reimbursement value was BRL 2,572 from SIGTAP code 04.06.05.001-5, in addition to 2 introducers (BRL 97). In both groups 1 and 2, the material costs were 1.5 times higher than the amounts reimbursed by the SUS.

## Discussion

In 2019, 6,471 electrophysiology procedures were performed by the SUS in Brazil. Considering data from the RECALL study,^13^ which showed that 5 out of every 100 patients with atrial fibrillation undergo ablation, and the Global Burden of Disease 2021,^14^ which estimates that 170,000 new cases of atrial fibrillation occur in Brazil annually, approximately 8,500 patients are expected to undergo atrial fibrillation ablation every year in Brazil. This number alone is higher than the total number of ablations performed in Brazil, considering all types of arrhythmias.

This study identified that highest number of procedures was performed in the South Region (48%), followed by the Southeast (31%) and Central-West (10%) Regions. This result does not align with the largest population concentrations in Brazil, which are found in the Southeast (41.8%), Northeast (26.9%), and South (14.7%) Regions. The North and Central-West Regions represent 8.5% and 8% of the population, respectively.

Nationwide, from the point of view of infrastructure, 49 of the 92 (53%) centers in Brazil had all the equipment necessary to perform cardiac arrhythmia ablation, which denotes a scarcity of facilities that are fully equipped for the procedure. Furthermore, the procedure requires experienced professionals, whose availability is still limited in middle- and low-income countries.^15,16^ Although specialized training is largely offered by public institutions, which reduces the need for external hiring, there are difficulties in retaining these specialists in the public system due to structural limitations, lack of remuneration compatible with the complexity of the work, and low professional stability.^17,18^ The centers accredited to perform electrophysiological procedures were concentrated in the South and Southeast Regions, even though all regions had the infrastructure and accredited centers. The other regions have a very small number of establishments that concentrate the procedures performed. Accordingly, the presence of centers with the availability of professionals and adequate equipment is indispensable for the safe performance of ablation procedures for arrhythmias and the sustainability of these services.

In this study, the North and Northeast Regions presented a lower number of qualified establishments per inhabitant. The North Region is located largely in the Amazon, a region characterized by a dense forest with large rivers and immense biodiversity, which includes sparsely populated cities. Diverse communities are located along river banks. Transportation to these communities occurs mainly by boat and depends on weather conditions. These characteristics hinder adequate access to medical care in the region, and this problem is aggravated by the lower socioeconomic level of the North Region compared to other Brazilian regions.^15^

Regarding the Northeast Region, a study analyzed hospitalizations and deaths due to conduction disorders and arrhythmias in Bahia. Their data showed that the state led the Northeast Region with 15,249 hospitalizations, corresponding to 32.1% of the total, and held second place in deaths due to arrhythmias, with 989 (25.8%).^19^ In Bahia, cardiac arrhythmias represent a considerable portion of hospitalizations and deaths in both public and private hospitals affiliated with the SUS.^19^ These data demand special attention from health authorities to reduce the morbidity and mortality associated with these conditions. Despite advances in the treatment of arrhythmias, associated deaths continue to represent a significant complication.^19^

It is worth highlighting the issue of material costs versus reimbursed amounts. This study showed that material costs (not considering other hospital costs and professional fees) are 1.5 to 4.8 times higher than the amounts reimbursed. In this context, a budget impact analysis using Brazilian data showed that, to the extent that procedure costs exceed the reimbursement issued by the SUS, the total costs, including hospital services and medical fees, require supplementary funding.^17^ This discrepancy can lead to material shortages and limit the number of procedures performed. Ablation for the treatment of arrhythmias is a complex procedure, as it depends on specialized techniques and advanced electrophysiological procedures performed in a referral center, requiring adequate remuneration to ensure the viability of the service within the SUS.^20^ This situation can leave a portion of the population without adequate care and dependent on drug treatment, even though medications are less effective in controlling arrhythmias compared to ablation treatment. Moreover, adverse events, low treatment adherence, and dropout rates are higher with medications.^21–25^

Cardiac rhythm disorders affect millions of people worldwide and are responsible for high rates of hospitalizations and diagnostic and therapeutic procedures.^26^ The global aging of the population contributes to the increased prevalence of arrhythmias, placing a significant burden on health systems and professionals responsible for these populations.^27,28^ This study has shown that, in order to maintain access to adequate arrhythmia treatment in Brazil, it is necessary to increase the number of properly equipped centers, reduce the need for patients to travel long distances, and adjust the reimbursement values for procedure costs.

### Limitations

The accuracy of data obtained from DATASUS (SIH/SUS) and CNES was not verified, based on the principle that the filling out of these forms followed the established recommendations and that each executing center correctly audited and validated the information they entered. Due to the use of secondary data, this study may present limitations associated with underreporting of procedures, inconsistencies in coding hospital records, and regional heterogeneity in information quality. Moreover, the discrepancies observed between the actual costs incurred and the amount reimbursed supports the hypothesis of underestimation related to limitations in the databases or the data collection methods. Furthermore, the analysis was restricted to the year 2019, the last year before the COVID-19 pandemic. This choice is justified by the exceptional variation in the number of hospitalizations observed during the pandemic period, which could bias analysis results. However, the choice of this time point poses limitations regarding the generalization of the results, given that the reimbursement value of electrophysiological procedures underwent an adjustment in 2022. Thus, the differences observed in this study may be greater than those that would be currently observed.

### Importance of the study

The Brazilian Ministry of Health/Secretariat of Science, Technology, and Innovation and the Economic-Industrial Complex of Health, through SECTICS/MS Ordinance Number 35 of May 27, 2025, published in the Official Gazette of the Union on May 29, 2025, made public the decision to incorporate, within the scope of the SUS, the contact force catheter and 3-dimensional electroanatomical mapping for radiofrequency ablation in the treatment of adult patients with complex cardiac arrhythmias.

## Conclusions

Within the scope of the SUS, there are significant regional disparities in the availability of electrophysiology services, especially in the North and Northeast Regions. The lack of equipped centers, the poor performance of several centers that do not conduct the minimum recommended number of procedures, and the low reimbursement rates for procedures represent critical challenges that need to be overcome in order to improve access to treatment of cardiac arrhythmias in Brazil.

## Data Availability

The underlying content of the research text is contained within the manuscript.

## *Supplemental Materials

Supplemental Material For additional information, please click here.
